# Exploring fulfillment of basic psychological needs in patients with dental anxiety: an investigation grounded in self-determination theory

**DOI:** 10.2340/aos.v85.46314

**Published:** 2026-06-29

**Authors:** Vibeke Kranstad, Geoffrey Colin Williams, Torill Helene Tveito, Vibeke Elise Ansteinsson, Anne Elisabeth Münster Halvari

**Affiliations:** a Oral Health Centre of Expertise in Eastern Norway, Oslo, Norway; b Faculty of Health and Social Sciences, University of South-Eastern Norway, Borre, Norway; c School of Business, University of South-Eastern Norway, Hønefoss, Norway; d Division of Cardiovascular Medicine, University of Michigan, Ann Arbor, MI, USA; e Department of Public Health Science, Institute of Health and Society, University of Oslo, Oslo, Norway

**Keywords:** Dental anxiety, cognitive behavioral therapy, motivation, self-determination theory, abuse

## Abstract

**Objective:**

This qualitative study aimed to investigate how patients with severe dental anxiety perceived cognitive behavioral therapy (CBT) delivered by interdisciplinary teams consisting of psychologists and dental personnel.

**Material and methods:**

Twelve individual semi-structured interviews were conducted at the Oral Health Center of Expertise in Eastern Norway, using an exploratory qualitative design. The analysis was guided by Braun and Clarke’s thematic analysis. Self-determination theory (SDT) was applied as the theoretical and analytical framework.

**Results:**

The qualitative analysis identified five themes shaping patients’ experiences with CBT and progress in managing dental treatment: *Establishing positive relationships with the psychologist and dental personnel*, *Experiencing enhanced competence in managing one’s anxiety, Discovering the opportunity to use one’s own capacities, Identifying factors nurturing plans for future dental visits*, and *Experiencing additional effects on quality of life*. These results underscore the importance of autonomy-supportive treatment that fulfills patients’ basic psychological needs (BPNs) for autonomy, competence, and relatedness, thereby fostering more autonomous motivation and desired behavioral changes.

**Conclusions:**

The findings indicate that CBT provided by interdisciplinary teams may benefit from adopting SDT as a framework, integrating autonomy-supportive principles to fulfill patients’ BPNs. This approach may enhance CBT’s patient-centered effectiveness and potentially improve patients’ quality of life.

## Introduction

Severe dental anxiety (DA), also known as dental phobia, is classified as a specific phobia in the Diagnostic and Statistical Manual of Mental Disorders, 5th ed. [1]. It involves an intense fear and avoidance of dental treatment, often associated with elevated levels of DA [2–4]. The term ‘DA’ is used throughout the article to refer to the full range of DA severity within the study sample. In the Nordic countries, approximately 10% of the population experiences DA, with 3%–5% classified as severe, consistent with other European findings [5–10]. High levels of DA are associated with poor oral health, social difficulties, negative effects on work, and reduced quality of life [11–14].

Traumatic events, such as psychological, physical, or sexual abuse, can significantly increase vulnerability to DA and worsen oral health [8, 15]. These observations align with findings from the Adverse Childhood Experiences (ACE) study [16], which demonstrated that multiple childhood adversities increase susceptibility to health risks in adulthood. Survivors of violence, sexual abuse, and individuals with severe DA constitute a particularly vulnerable group, often experiencing comorbid conditions, such as post-traumatic stress disorder, dissociative disorders, and depression [17]. These comorbidities make the treatment of DA complex [8, 18, 19] and likely affect how these vulnerable patients approach and adapt to treatment of DA. Violence, sexual abuse, and torture are major risk factors for DA and often trigger trauma responses in dental treatment settings [19–25]. It has been proposed that child sexual abuse survivors may experience DA as a long-term trauma effect [19]. With high rates of violence and sexual abuse in Europe [26, 27] and increasing migration from conflict zones [28, 29], certain populations may require increased attention and tailored interventions for DA.

Since 2012, the Norwegian public dental service has provided the TADA (i.e. Torture, Abuse, and/or DA) service for those who have experienced torture, violence, sexual abuse or have dental phobia [30]. This free service offers cognitive behavioral therapy (CBT) and exposure therapy (ET), primarily provided by dentists with support from a psychologist and a dental team. CBT is a structured, evidence-based psychotherapy that focuses on identifying and modifying negative thought patterns and behaviors to improve emotional regulation and develop healthier coping mechanisms. After completing CBT, patients transition to a specialized team focused on oral health restoration.

CBT is the preferred treatment for DA, helping patients overcome anxiety-related challenges, which may, in turn, improve oral health [31–33]. CBT encompasses a range of techniques widely used in modern counseling. Rather than being a single method, CBT serves as an umbrella term for diverse approaches [34]. Some psychological aspects of anxiety and motivation in patients attending the TADA service remain underexplored, and the effectiveness of CBT compared with other approaches is uncertain [35, 36]. Hence, further research is needed to clarify CBT’s key elements and address its limited focus on motivation, particularly the psychological need for autonomy [37].

**Figure 1:**
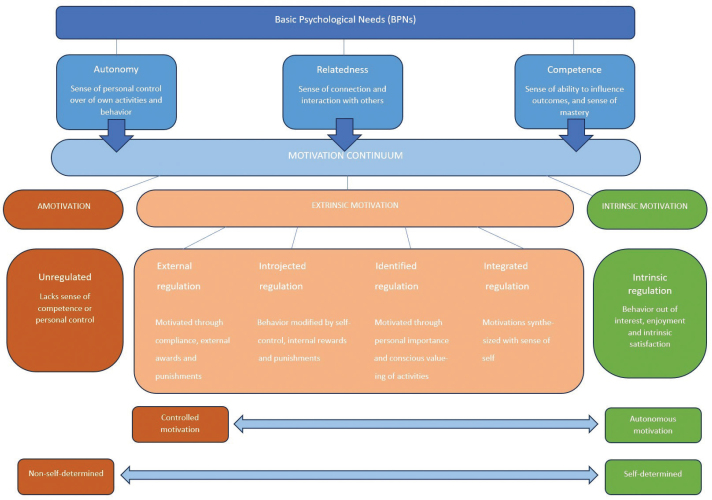
The motivation continuum of Self-Determination Theory. Adapted from Ryan and Deci [39].

According to Self-Determination Theory (SDT) [37–39], motivation is not only about the extent to which patients want to change but also about the quality of their motivation, which plays a significant role in the change process [34]. As illustrated in Figure 1, the SDT motivation continuum ranges from non‑self‑determined (amotivation and controlled forms of extrinsic motivation) to fully self‑determined, intrinsic motivation. SDT posits that when individuals’ basic psychological needs (BPNs) for autonomy, competence, and relatedness are satisfied, they are more likely to engage meaningfully in therapy. In this context, patient engagement reflects autonomous motivation, which arises when patients personally endorse the value of therapeutic change. Patients with psychological vulnerabilities, such as comorbid anxiety, trauma, or previous negative dental experiences, may experience ambivalence or resistance to change [34], potentially undermining both engagement and therapeutic outcomes in CBT. It has therefore been suggested that CBT could benefit from the integration of supplemental elements aimed at enhancing patient motivation and engagement [34, 40]. For example, a study by Britton et al. [41] found that supplementing CBT for suicide prevention with Motivational Interviewing (MI) techniques enhanced engagement, as MI strategies actively supported patients’ BPNs. Other studies [42, 43] also support the view that integrating MI, a method that aligns with SDT principles by fostering patient choice, empathy, and intrinsic motivation, can reduce resistance and enhance engagement in traditional CBT contexts.

This study primarily focuses on patients’ experiences of fulfilling their BPNs during CBT, while also exploring certain instances where these needs appeared to be unmet.

### Using self-determination theory as the theoretical and analytical framework

SDT [37–39] is a well-established framework for understanding human motivation. It was selected as the theoretical foundation for this study due to its emphasis on the universality of human BPNs, its explanation of the internalization process, and its relevance across health care contexts [37]. SDT comprises six mini-theories, including Basic Psychological Needs Theory (BPNT), which identifies three essential needs: *autonomy* (i.e. the need to act in accordance with one’s values and volition), *competence* (i.e. the need to interact effectively with the environment), and *relatedness* (i.e. the need to feel accepted and valued by others). Research suggests these three needs are fundamental across individuals and cultures [44]. SDT posits that motivation and self-regulation flourish when the BPNs are satisfied and are undermined when these needs are frustrated or thwarted [38, 45–47]. In this framework, *self-regulation* refers to an individual’s ability to manage thoughts, emotions, and behaviors in line with personal values, rather than responding to external pressures. *Autonomous motivation* – which includes intrinsic motivation and internalized forms of extrinsic motivations, such as identified and integrated regulation – develops through the adoption and integration of personally meaningful values and goals. SDT differentiates types of motivational quality along a continuum: At one end are *external* and *introjected regulations*, which are more controlled types of motivation driven by external pressure or feelings of guilt. Conversely, *identified* and *integrated regulation* represent more autonomous forms of motivation, closely aligned with an individual’s personal values. *Amotivation* is described as a complete lack of motivation to act [38]. SDT claims that social environments play a key role in the process of internalization, often influencing how motivations are integrated into a person. Research indicates that when contexts support BPNs, individuals are more likely to internalize autonomous forms of motivation [37].

In health care, fostering BPNs through *autonomy-supportive practices* entails offering choices, providing meaningful rationales, and validating patient perspectives. Such practices have been shown to enhance patient engagement and facilitate long-lasting behavior change [48–50]. Specifically, patients with more *autonomous self-regulation* and motivation (i.e. identified and integrated regulation) are more likely to willingly engage in emotionally challenging treatments, such as CBT for DA, and to sustain long-term self-regulated behaviors related to DA and oral health [48, 51, 52]. A meta-analysis by Ntoumanis et al. [53] found that SDT-based interventions that enhance need support and autonomous motivation improve health outcomes. Accordingly, dental care environments that actively support patients BPNs may help TADA patients engage more effectively in treatment and reduce DA. [54, 55].

This study aimed to explore how patients perceived CBT provided by TADA teams, focusing on which elements they found helpful. Additionally, the study employed the BPN framework of SDT [37–39] to interpret participants’ perceptions of support for autonomy, competence, and relatedness during treatment.

## Material and methods

This study utilized an exploratory qualitative design with an abductive approach. Individual semi-structured in-depth interviews were undertaken from October 2020 until June 2021.

### Participants and recruitment

All patients undergoing CBT in the TADA service at the Oral Health Center of Expertise in Eastern Norway (OHCEEN), Oslo, during the study period, were invited by TADA team members to participate in the study. Twelve participants (4 men) between 28 and 68 years old were recruited. Table 1 provides an overview of the sample characteristics. The participants had attended a mean of 14.4 treatment sessions (range 6–29 sessions).

**Table 1:** Participants’ descriptives.

Category	Variable	*n*
Gender	Female	8
	Male	4
Trauma experience	Violence	2
	Sexual abuse	6
	Torture	0
	Refugee	1
	None	3
Dental phobia at referral	Yes	10
	No	2
Education	Higher 3 years or more	6
	No higher	6
Treatment sessions	1–10	5
	11–20	5
	21–30	2

The inclusion criteria matched those required for participation in the TADA service: Adults who (1) have experienced torture, abuse, or violence, and/or display clinical symptoms of current dental phobia, (2) are turning 21 that year, (3) are willing and able to commit to a treatment plan developed with an interdisciplinary team, and (4) understand the rationale and principles of the proposed treatment. Additional criteria included: (5) fluency in Norwegian and being under the age of 65. Exclusion criteria included: (1) patients not meeting the TADA service inclusion criteria as assessed by the psychologist, (2) those with organic disorders such as dementia, delirium, or severe memory issues, and (3) those with cognitive/linguistic abilities consistent with intellectual disability. We retained one participant over 65 years old who was included in the study by the TADA team.

Patients who expressed interest in participating in the study received information about the project both orally and in writing from the TADA teams. The first author called the participants to offer study details and schedule the interview, and provided an initial introduction to the researcher. All participants attended their scheduled interview appointments and signed the informed consent form digitally on an iPad using a webform service hosted by the University of Oslo (UiO) (Nettskjema) before the interviews.

### Data collection

The first author conducted all interviews face-to-face at the OHCEEN. The interviews were held in a welcoming and thoughtfully arranged room and lasted between 1 and 2 h. The interviews were audiotaped using an iPad with a recording function integrated into the webform service, with immediate transfer to a safe server (Services for sensitive data [TSD, UiO]). The first author transcribed all interviews verbatim. The data collection continued until the need for sufficient ‘information power’ was satisfied [56], which was achieved at 12 participants. The interview guide (Table 2) was constructed by the first and last authors, based on the literature, their prior knowledge, clinical practice, and experiences from the first author’s work in a TADA team and Halvari et al.’s research [52, 54, 55] using BPNT within SDT [38, 39]. The interview guide consisted of 14 questions on how the patients perceived the CBT conducted and the people who constituted the TADA teams. The function of the interview guide was more of a checklist to ensure that all themes were elicited, rather than providing strict guidance for the interviews.

**Table 2:** The interview guide.

Q1 What led you to choose anxiety treatment through the TADA service?
Q2 How did you find the anxiety treatment provided by the TADA team?
Q3 What aspects of your relationship with the dentist, dental hygienist, dental health secretary, and psychologist did you find positive or negative during the treatment? → This question was designed to address the basic psychological **need for relatedness**, as described in Self-Determination Theory (SDT), by encouraging participants to reflect on the interpersonal qualities of the treatment setting and how these may have influenced their engagement.
Q4 How did you experience the emotional support from the dentist during the treatment? → This question was intended to explore the need for **relatedness**, with a particular emphasis on the emotional and relational quality of the therapeutic alliance. It invited participants to reflect on whether they felt understood, cared for, and supported, which are key aspects of relatedness as conceptualized in SDT.
Q5 How did you experience having a say in decisions during your treatment? → This question was formulated to explore the need for **autonomy**, as defined in SDT. Supporting autonomy involves fostering a sense of agency, and this question helped examine whether such conditions were present in the therapeutic setting. Q6 What were your thoughts on the time allocated to you during the treatment, both in each session and overall? → This question was meant to yield insight into **relatedness;** being respected and supported by the therapist, and **autonomy**; having space to express one’s own needs and experiences without time pressure.
Q7 How well did you feel the dentist addressed your need for competence (information, understanding, and mastery) during the treatment? → This question was designed to explore the **need for competence**, as described in SDT. It aimed to assess whether participants felt that the dental team provided them with sufficient information, clear explanations, and appropriate support to help them understand and manage their CBT. Fulfilling the need for competence involves feeling effective and capable in one’s actions. This question allowed us to examine whether the therapeutic environment supported the participants in developing a sense of mastery and confidence in coping with dental anxiety but also with more general anxiety, particularly among those with trauma histories.
Q8 How did you handle your emotions and physical reactions during the treatment? → This question intended to invite participants to reflect on their sense of effectiveness and capability in managing difficult internal experiences. Feeling that one can regulate emotional and physiological responses during a challenging situation (i.e. CBT/dental treatment) is directly tied to **competence** fulfillment in SDT. Q9 How did you assess your resilience and ability to complete the anxiety treatment? → This question addresses the **competence** need in SDT by encouraging participants to reflect on their skills, capabilities, and confidence in managing the CBT. It supports the sense of competence need by prompting them to evaluate their progress, self-efficacy, and ability to overcome challenges, reinforcing their feelings of mastery and effectiveness.
Q10 How did you find the experience of completing the anxiety treatment, and what would you say was the most important thing you learned during the anxiety treatment?
Q11 How do you believe your participation in anxiety treatment within the TADA service has impacted your quality of life? Q12 How do you perceive the transition from the TADA team to the team that will complete your dental treatment?
Q13 What do you anticipate will be the most challenging aspect of the dental restoration phase within the TADA service?
Q14 How did you find the experience of this interview?

TADA: torture, abuse, and/or dental anxiety; CBT: cognitive behavioral therapy.

*Note:* Naturally arising follow-up questions such as ‘how did you experience being given opportunities and choices during the treatment? (Q5)’ were asked if the questions already asked were not adequately understood or if themes were not sufficiently elaborated.

### Analysis and data management

The qualitative data were analyzed using thematic analysis as outlined by Braun and Clarke [57], with SDT [37–39] serving as the analytical framework. Thematic analysis is a flexible, six-phase method for identifying, analyzing, and reporting patterns (i.e. themes), both congruent and divergent, within qualitative data. All transcripts were read by the first and last authors with open-minded awareness, to gain a holistic view and an overall first impression of how the participants perceived the CBT provided by the TADA teams. Initial coding enabling separation, sorting, and synthesis of the data was performed by the first author [57]. Codes with similar content were organized into the same categories. The coding reflected the ‘data- and theory-driven’ nature of the themes [58]. The transcripts and codes were reviewed by the first and last authors to check that each code fitted the transcriptions. At all stages of the analysis, the themes and sub-themes were reviewed using the transcripts and the reflective journal, including memo notes, to ensure that the generation of themes/sub-themes accurately reflected the narratives they represented. The text analysis program NVivo 12 Pro Software (QSR International, Daresbury, UK) was used to organize codes and themes throughout the analysis.

### Ethical considerations

The study was conducted in accordance with the 2013 Declaration of Helsinki [59], and the participants were informed of their rights consistent with this. All interviews were undertaken with the understanding and written consent of each participant. For traumatized individuals, recalling painful experiences may trigger unwanted reactions. Hence, psychological support was offered if required, but none of the participants sought it. The study was approved by the Regional Committee for Medical and Health Research Ethics in South-East Norway (project no: 135973) and the Norwegian Agency for Shared Services in Education and Research (project no: 125842) before the study started.

## Results

During the interviews, the participants expressed satisfaction with the CBT phase of the TADA service and shared diverse experiences, both positive and negative. Their narratives centered on their motivation for behavioral change, alleviating DA, and how the TADA teams met their needs for autonomy, a trusting relationship, and dental treatment management. Insights were gained into what participants valued in their experiences with the anxiety treatment and how the fulfillment of their psychological needs was supported. The resulting themes and sub-themes are outlined in Table 3, with five overarching main themes capturing the content of all sub-themes. Table 4 provides a detailed overview of how participants perceived the fulfillment of their BPNs. This study utilizes the framework of BPNs from SDT [37–39] to explore how CBT can be tailored to better support the needs of patients in the TADA service. Participants also reported improved quality of life and enhanced life prospects as a result.

**Table 3:** Themes and sub-themes reflecting participants’ experiences with Cognitive Behavioral Therapy.

Themes	Sub-themes
Theme 1: Establishing positive relationships with the psychologist and dental personnel	Supportive clinical environment Sense of choice, influence, and self-control in treatment Not feeling rushed through the treatment course
Theme 2: Experiencing enhanced competence in managing one’s anxiety	Mental presence and being in control of one’s own anxiety reactions Setting boundaries for oneself and expressing one’s needs Exposure to real dentistry scenarios
Theme 3: Discovering the opportunity to use one’s own capacities	Training on using one’s own resources Motivation for anxiety treatment Self-acceptance and being in autonomous control reduce shame
Theme 4: Identifying factors nurturing plans for future dental visits	Preparing a coping plan in collaboration with the dentist Having trust in handling one’s own anxiety Experiencing a sense of normality by having plans for future dental visits
Theme 5: Experiencing additional effects on quality of life	Being more aware and distinguishing the present from the past Increased self-confidence and a general feeling of being self-determined Genuine expression of self-promotion and personal growth A brighter tomorrow: A new vision of the future

**Table 4:** Thematic examples of Basic Psychological Needs (BPNs) fulfillment as interpreted from participant narratives.

Theme	Representative participant quotations illustrating need fulfillment	How the need was fulfilled – Researchers’ interpretation
Theme 1: Establishing positive relationships with the psychologist and dental personnel	*Relatedness*: ‘I felt very seen, I felt there was a lot of room to talk about what was difficult for me about dental treatment but also draw some parallels to how dental treatment can be compared to other things in life in a way. I felt she had a lot of time for me…’ (ID2)	Participants felt seen, listened to, and emotionally supported in a meaningful and individualized therapeutic relationship.
	*Autonomy, competence, and relatedness:* ‘It was the fact that I was constantly given the certainty and security that I was the one who had to manage things here, and that I had to speak up when things got uncomfortable.’ (ID1)	Participants felt empowered to manage their treatment and express their feelings (reflecting competence), reinforcing a sense of agency and autonomous self-direction (reflecting autonomy). The ‘certainty and security’ provided by the therapist signals relatedness.
	*Relatedness, and competence (relational):* ‘I felt that there was enough time for the reactions that would eventually come, it was as if nothing was rushed. I felt I was taken very seriously, which I have not experienced when I have been to previous dentists.’ (ID2)	Participants experienced a respectful, supportive, and unrushed clinical setting, which supported the need for relatedness by fostering trust and engagement in the therapeutic process. Participants appeared to feel effective in managing relationships and navigating social interactions with the personnel (relational competence).
Theme 2: Experiencing enhanced competence in managing one’s anxiety	*Competence:* ‘… it was becoming aware of my body’s own signals that now I am entering a higher level of anxiety. And being able to feel on my body that now something is being triggered, and be aware of it, and then be able to say that “now I need a break”.’ (ID12)	Participants experienced an increased sense of competence by learning to recognize bodily signs of anxiety and respond more effectively, demonstrating improved self-awareness and mastery in managing their emotional reactions during treatment.
	*Autonomy, and competence:* ‘… she [the dentist] was also very concerned about boundaries, my boundaries. And that I should recognize “what is important to me?”. […] It may sound very banal, but it was very new to me, because suddenly I was supposed to acknowledge “what do I need?”.’ (ID4)	Participants experienced autonomy support through the dentist’s emphasis on personal boundaries and encouragement of self-awareness, which appeared to help them recognize and express their own needs (reflecting autonomy). This, in turn, seemed to foster a greater sense of agency and enhance their ability to regulate anxiety (reflecting competence).
	*Competence, and relatedness:* ‘For example, the cotton roll, it’s completely loose, and then he [the dentist] showed in a mirror and such, that it is placed very far up here [under the upper lip], and I can feel that it is firmly stuck, that helps.’ (ID9)	Participants felt their competence in managing anxiety was supported through clear, tangible information and demonstrations, which seemed to enhance their understanding and sense of agency and mastery. Relatedness appeared fostered by the dentist’s attentive and reassuring communication, which built trust and a sense of being cared for.
Theme 3: Discovering the opportunity to use one’s own capacities	*Competence, autonomy, and relatedness:* ‘..so, she was very surprised about how much competence I had, and to which degree I was able to see my own situation through that course of treatment and relate all the knowledge. […] Whereas here, the knowledge I brought with me from before, it was seen as a resource. […] She [the dentist] was interested in hearing my reflections on the situations. And then I was seen more as a person, and that … she gave me more hope.’ (ID4)	Participants’ experienced fulfillment of competence through being recognized for their abilities and prior knowledge, autonomy through being invited to reflect and make meaning of their own experience, and relatedness through feeling seen, respected, and emotionally supported.
	*Autonomy:* ‘I didn’t feel that there was anyone pushing me forward, I felt that it was a lot myself pushing me forward. And it’s probably better than someone else doing it. […] You get more control [autonomous self-regulation] then in a way.’ (ID6)	Participants experienced a sense of volition, personal initiative, and psychological ownership of the treatment process. This suggests that their motivation was more autonomously regulated and that the psychological climate of the clinical environment supported, rather than pressured, their engagement.
	*Competence, and autonomy:* ‘I feel shame towards myself, and perhaps a little bit towards society, because this is such a normal adult thing that you should be able to manage, like going to the dentist, which is taking care of your teeth, and following up on such basic, banal things. And then feeling that you can’t master it, at least, it makes me ashamed. And knowing that now you’re starting to master it, …to manage it, that was motivating.’ (ID12)	Illustrates the fulfillment of competence, as it describes a shift from shame and incapability to a motivating sense of mastery over a previously overwhelming task. Fulfillment of autonomy, as the change appears self-endorsed, personally meaningful, and intrinsically valuable.
Theme 4: Identifying factors nurturing plans for future dental visits	*Autonomy, competence, and relatedness:* ‘..then it’s in my medical record [the coping plan], and then the dentist will read it, and take his time for it before we start talking, and then I don’t have to deal with Pandora’s box at all. It can somehow be placed far away, and they can recognize what activates me, and know what helps me best then. There will be a completely different control [autonomous self-regulation] in that situation.’ (ID4)	Illustrates the fulfillment of autonomy by enabling participants to feel a sense of volition and agency through a pre-agreed coping plan, supporting autonomous self-regulation as they experience ownership over the process. It also supports competence and relatedness by fostering a sense of preparedness and ensuring they are met with understanding and care.
	*Competence, and autonomy:* ‘I might be able to relate to it [the anxiety]. That must be almost a tool, that I relate to it.’ (ID6)	Illustrates the fulfillment of competence, as participants describe gaining a sense of mastery in recognizing and relating to their anxiety as a usable tool. It also reflects autonomy, as it demonstrates personal ownership of this coping approach.
	*Autonomy, competence, and relatedness:* It will be a process of finding the right one [dentist] and such, but I think it’s something I’m going to do. […] … that I will go to the dentist regularly. […] … a bit like to feel a bit normal too, because that’s what ordinary people do. […] So, I just want to get it as a routine in a step closer to that normality then.’ (ID2)	Reflects the fulfillment of autonomy, as participants express a self-endorsed intention to maintain regular dental care. Also supports competence, through a growing confidence in managing this routine, and suggests relatedness, by expressing a desire to feel more normal and connected to social expectations.
Theme 5: Experiencing additional effects on quality of life	*Competence, and autonomy:* ‘I’ve noticed quite a lot ... big transfer value to other things in life, that I can manage to stand in discomfort in the present without getting completely lost in the past then, and I can notice that in other arenas in life, and it is very good ... I’ve had fewer memories of trauma and, sort of, fewer such panic attacks, and it’s often then that my thoughts have taken me back in time and I’ve sort of relived several such old traumas, less of that. More, what do you call it … mindfulness, or more like a here-and-now experience.’ (ID2)	Illustrates the fulfillment of competence, as participants describe increased ability to manage emotional discomfort and trauma-related symptoms, with perceived progress extending into other areas of life. It also suggests growing autonomy through enhanced self-regulation and mindfulness, reflecting a greater sense of volition and mastery over emotional experiences.
	*Competence, relatedness and autonomy:* ‘My cohabitant notices it quite well. He has been surprised at how little anxiety there has been lately. And notice that I have much more fighter-like attitude then. […] And that overwhelming insecurity about everything that was unknown was also very much more present before, than I feel it is now.’ (ID2)	Reflects the fulfillment of competence through increased mastery over anxiety, and relatedness through supportive social acknowledgement by a significant other (i.e. the cohabitant). Feelings of self-regulation and self-control reflect autonomy.
	*Competence, autonomy, and relatedness:* ‘I became freer, I became more self-confident, I became more unbothered. And when you get more unbothered, you remember things. […] Yeah, just as if my head wasn’t…. what can I say, wasn’t filled with cotton. […] But it’s just like all these years my brain has been paralyzed; your emotional life has been paralyzed. And then it was just as if someone [during CBT] opened it up. I’m not so afraid of what happens in the future. […] My thoughts have become clearer. I don’t have those dark trains of thought that I had before.’ (ID3)	Illustrates the fulfillment of competence through increased self-confidence and emotional clarity, suggesting improved ability to manage thoughts and feelings. Also reflects growing autonomy, as participants express a sense of freedom and reduced fear about the future. A supportive and meaningful therapeutic relationship, which considered the support of all three BPNs may have contributed to this transformation (e.g. ‘as if someone opened it up.’), highlighting the value of relatedness support.

All quotes from the Results section are included to illustrate how specific BPNs are fulfilled and reflected in the data.

### Theme 1: Establishing positive relationships with the psychologist and dental personnel

Several aspects of the participants’ experiences contributed to their open and favorable relationship with the TADA team members. The participants recognized and appreciated specific psychological aspects of their clinical environments, such as feeling that therapy sessions were well spent, conducted on their terms, and that they were seen for who they truly were. This supportive psychological environment fostered a sense of relatedness among the participants and made them feel safeguarded:

‘I felt very seen, I felt there was a lot of room to talk about what was difficult for me about dental treatment but also draw some parallels to how dental treatment can be compared to other things in life in a way. I felt she had a lot of time for me, that the time set aside was somehow well managed and was very much on my terms. Yes, and also, I felt that she was very knowledgeable.’ (ID 2)

The participants recognized the TADA teams as highly skilled in managing trauma and anxiety, a competence that positively influenced their own experience. Some preferred being alone with the dentist for greater autonomy and trust, while others expressed feeling more secure with both a dentist and dental health secretary present.

The participants described being involved in decision-making during anxiety treatment, suggesting the development of autonomy, competence, predictability, and relatedness. This involvement seemed to have enhanced their self-regulation, fostering more autonomous motivation and thus encouraging them to develop skills for managing dental treatment. They also conveyed that it strengthened their role as proactive members of the treatment team by encouraging them to express their needs:

‘It was the fact that I was constantly given the certainty and security that I was the one who had to manage things here, and that I had to speak up when things got uncomfortable.’ (ID 1)

Several participants initially found it unfamiliar to express their needs but felt safer and developed a positive relationship with the TADA team when given the opportunity. Most participants reported that being allowed to influence their treatment, rather than strictly following a specific method, supported their sense of autonomy. This flexibility appeared to make them feel safe and nurtured a more secure attachment to the TADA team. A few participants could not communicate their wishes during the treatment, reporting that they felt controlled or pressured by the therapist. One possible explanation is that they felt intimidated by authority figures and unaccustomed to expressing their opinions and needs. The essential competence to express their feelings and needs did not appear to be fully developed or achieved during CBT.

The participants noted that the ample time allotted for each treatment session made them feel well cared for according to their individual emotional and psychological needs, allowing them to make their own choices. This approach appeared to foster a greater sense of autonomous self-regulation among several participants. They expressed feeling heard and seen, which strengthened their relationship with their therapist and indicated a sense of autonomy and self-endorsement:

‘I felt that there was enough time for the reactions that would eventually come, it was as if nothing was rushed. I felt I was taken very seriously, which I have not experienced when I have been to previous dentists. […] That somehow there won’t be time and there won’t be any understanding then. I feel that very much here [there’s time and understanding]. And that it is sort of done on my terms.’ (ID 2)

One participant emphasized that there would be limited progress and new learning if she were to decide the treatment pace the whole time. She expressed a desire to advance in her treatment to achieve her health goals and acknowledged that this would require input from the TADA team. A few participants described how the lack of more individualized, mastery- and capacity-focused support within CBT limited their ability to experience a sense of need fulfillment.

### Theme 2: Experiencing enhanced competence in managing one’s anxiety

Different aspects of learned competencies that contributed to improving their sense of mastery in managing anxiety during treatment were described by the participants. They shared that by staying present and mindful during their treatment, they became more attuned to and recognized their physiological reactions to increased anxiety levels. This heightened mindfulness appeared to facilitate more effective situational self-regulation:

‘Yes, so there was such a change in mindset, on the one hand, and on the other hand, it was becoming aware of my body’s own signals that now I am entering a higher level of anxiety. And being able to feel on my body that now something is being triggered, and be aware of it, and then be able to say that “now I need a break”. Because I just used to disconnect [mentally], and then I just let the dentist do what he needed to do, without participating in the situation.’ (ID 12)

Some participants had previously associated dentistry with violence or sexual abuse due to a lack of mindfulness during dental procedures. Through CBT, they acquired new skills to manage their anxiety by staying mentally present during therapy. This newfound competence was interpreted as having enabled participants to better manage their anxiety responses, develop a sense of mastery, and self-regulate more effectively. As a result, they appeared more able to engage fully and authentically with the treatment.

Participants reported that pushing themselves too hard during treatment, without attending to their individual needs, led to dissociative experiences, which in turn seemed to hinder the development of new skills and competence. They shared that by practicing mindfulness and recognizing when their anxiety became overwhelming, they were able to avoid dissociation and further develop their self-regulation.

The participants mentioned being encouraged and allowed to make decisions during treatment, which was understood to support their sense of autonomy. Collaborative discussions with the dentist about how to adapt CBT to their individual needs appeared to foster their sense of relatedness. These supportive interactions seemed to promote more active engagement in therapy and a stronger sense of benefit. Being given time to reflect on their own needs appeared to facilitate the development of trust in the TADA team. Through these processes, participants seemed to become more self-determined, along with an increased sense of competence:

‘… she [the dentist] was also very concerned about boundaries, my boundaries. And that I should recognize “what is important to me?”. […] It may sound very banal, but it was very new to me, because suddenly I was supposed to acknowledge “what do I need?”.’ (ID 4)

The participants explained that training during real routine dental procedures and in actual treatment settings, enhanced their perceptions of improved competence in managing their anxiety. This experience gave them more confidence in their ability to master challenges and seemed to foster autonomous motivation to reduce their DA:

‘And then there is experience. For example, the cotton roll, it’s completely loose, and then he [the dentist] showed in a mirror and such, that it is placed very far up here [under the upper lip], and I can feel that it is firmly stuck, that helps. But telling me there’s no room for it in my throat, or something like that, doesn’t help much. So, it’s doing things for real, that helped.’ (ID 9)

Some participants described the ET as overly simulated and lacking realism. We interpreted this as potentially limiting their development of genuine and helpful competence in managing their DA.

### Theme 3: Discovering the opportunity to use one’s own capacities

The third theme explored how participants felt more vital and empowered through support and encouragement, discovering how to utilize their own abilities. This process seemed to lead to greater autonomous motivation, self-acceptance, and a feeling of more appropriate and important competence, thereby fostering additional self-determined behavior. Participants reported that receiving social and BPN support helped them uncover inner conscious resources and abilities, enabling them to better regulate their emotions and enhance self-regulation through self-endorsement:

‘… she also said to me, “you can do a lot”, she said in conclusion, “you are able to” … so, she was very surprised about how much competence I had, and to which degree I was able to see my own situation through that course of treatment and relate all the knowledge. […] Whereas here, the knowledge I brought with me from before, it was seen as a resource. […] She [the dentist] was interested in hearing my reflections on the situations. And then I was seen more as a person, and that … she gave me more hope.’ (ID 4)

The participants highlighted that the risk of pushing themselves too hard during treatment could trigger re-traumatization or dissociation, undermining its effectiveness. Many also emphasized the importance of a therapist who could recognize these risks and assist in co-regulating their emotions and anxiety.

The motivation for treatment was indicated by most participants to be high, often driven by the dual goals of restoring their teeth and reducing DA. This motivation was evident in various ways throughout the treatment and appeared to be supported by the extent to which their needs for relatedness, autonomy, and competence were met. Several participants shared a more conscious and self-regulated approach to managing their anxiety, demonstrating a higher degree of autonomous motivation:

‘I didn’t feel that there was anyone pushing me forward, I felt that it was a lot myself pushing me forward. And it’s probably better than someone else doing it. […] You get more control [autonomous self-regulation] then in a way. […] I think maybe patients are a little dissimilar. I was very motivated for this [CBT]. Extremely motivated, and I think it kind of helps. If I had just come and expected someone to fix me or something, I’m not sure if it would have worked particularly well, really. […] then maybe I wouldn’t have related to it [the anxiety] that way either.’ (ID 6)

The participants realized that they could not ‘be fixed’ by the therapist, but rather needed to engage themselves in the therapeutic process, demonstrating more autonomous motivation. The attitude of the TADA teams and the autonomy-supportive atmosphere were reported to contribute to this experience. Some participants expressed controlled motivation during treatment, being more extrinsically motivated by external factors, such as dental pain, shame, poor quality of life, and being a bad role model to their children. Several shared that this treatment option was their last resort to alleviate their DA.

The participants emphasized that during CBT, accepting that there were reasonable explanations for their difficulties in receiving dental treatment allowed them to experience increased self-acceptance and provided an opportunity to develop more autonomous self-regulation. This acceptance seemed to nurture their need for competence and replace feelings of shame with a sense of self-regulation over the treatment process:

‘I feel shame towards myself, and perhaps a little bit towards society, because this is such a normal adult thing that you should be able to manage, like going to the dentist, which is taking care of your teeth, and following up on such basic, banal things. And then feeling that you can’t master it, at least, it makes me ashamed. And knowing that now you’re starting to master it, …to manage it, that was motivating.’ (ID 12)

### Theme 4: Identifying factors nurturing plans for future dental visits

Several treatment factors were identified as influencing the participants’ plans for future dental visits, including preparing a coping plan, effective anxiety management, and cultivating a sense of normality. Implementing a coping plan for the new dentist to follow during future dental visits allowed participants to anticipate self-regulation during treatment. This approach appeared to assure them that the new patient-dentist relationship was safe and helped eliminate the need to repeatedly explain their difficulties in managing dental treatment:

‘I was so happy that I started to cry, because then I won’t have to talk about it again, then it’s in my medical record, and then the dentist will read it, and take his time for it before we start talking, and then I don’t have to deal with Pandora’s box at all. It can somehow be placed far away, and they can recognize what activates me, and know what helps me best then. There will be a completely different control [autonomous self-regulation] in that situation.’ (ID 4)

A few participants acknowledged that they became overly reliant on the coping plan rather than drawing on their inner autonomous motivation. This was interpreted as a reflection of insufficient development of competence in managing escalating anxiety during CBT. As a result, participants suggested having more sessions involving real dentistry training.

Participants’ accounts suggested that their needs for anxiety competence, autonomy, and relatedness were supported during treatment. In combination with the experience of directly confronting their anxiety, this appeared to contribute to a growing sense of trust in their ability to manage potential increases in DA during future dental visits:

‘I might be able to relate to it [the anxiety]. That must be almost a tool, that I relate to it.’ (ID 6)

A few participants appeared to lack autonomous motivation, as reflected in their limited cognitive reappraisal of dental treatment. This seemed to obstruct participants’ ability to fully engage in the therapeutic process, thereby hindering the development of effective anxiety management skills. It suggests that they may not have fully integrated the rationale for treatment in a more autonomously regulated manner.

Several participants expressed their intention to attend dental appointments regularly after completing the TADA treatment, reflecting a long-awaited return to a healthier and more self-regulated approach to dental care. They recognized that this would require investing time and effort to find the right dentist with whom they could establish a confident and enduring relationship:

‘It will be a process of finding the right one [dentist] and such, but I think it’s something I’m going to do. […] … that I will go to the dentist regularly. […] … a bit like to feel a bit normal too, because that’s what ordinary people do. […] So, I just want to get it as a routine in a step closer to that normality then.’ (ID 2)

Some participants had not given much thought to their future dental care visits, indicating less integrated autonomous motivation. However, they acknowledged the importance of having such forthcoming plans to ensure the lasting benefits of their anxiety treatment.

### Theme 5: Experiencing additional effects on quality of life

The participants highlighted that participating in the TADA treatment positively impacted their personal lives by increasing their mindfulness, boosting their self-confidence and self-advocacy, and fostering a more hopeful outlook for the future. Being more present and less focused on past traumas was noted by the participants and helped improve their sense of autonomous self-regulation. By focusing on the present and distancing themselves from past trauma, participants appeared better able to develop more self-determined behaviors, as external distractions and emotional blockages were reduced:

‘I’ve noticed quite a lot ... big transfer value to other things in life, that I can manage to stand in discomfort in the present without getting completely lost in the past then, and I can notice that in other arenas in life, and it is very good ... I’ve had fewer memories of trauma and, sort of, fewer such panic attacks, and it’s often then that my thoughts have taken me back in time and I’ve sort of relived several such old traumas, less of that. More, what do you call it … mindfulness, or more like a here-and-now experience.’ (ID 2)

Participants’ experiences of a safe and trusting relationship with the TADA team, coupled with a sense of self-determination and active engagement in their treatment, reflect a progression toward more autonomous self-regulation. This enhanced their confidence not only in managing their DA but also in other areas of life:

‘My cohabitant notices it quite well. He has been surprised at how little anxiety there has been lately. And notice that I have a much more fighter-like attitude then. […] And that overwhelming insecurity about everything that was unknown was also very much more present before, than I feel it is now.’ (ID 2)

Many participants reported that after the CBT, they felt more capable of expressing their authentic selves and more confident in stating their opinions. This was interpreted as reflecting increased autonomy, competence, and confidence in mastering and managing their anxiety, which, in turn, appeared to reduce their concern with others’ opinions and judgments:

‘I became freer, I became more self-confident, I became more unbothered. And when you get more unbothered, you remember things. […] Yeah, just as if my head wasn’t…. what can I say, wasn’t filled with cotton. […] But it’s just like all these years my brain has been paralyzed, your emotional life has been paralyzed. And then it was just as if someone [during CBT] opened it up.

I’m not so afraid of what happens in the future. […] My thoughts have become clearer. I don’t have those dark trains of thought that I had before.’ (ID 3)

One participant said he had become a more complete version of himself. Most participants reported having a more optimistic view of the future, both regarding dental treatment and life changes. They mentioned feeling less anxious and depressed, more mindful, and experiencing more autonomous self-regulation. This was reported to help them take greater ownership of various aspects of their lives, fostering a sense of personal growth. These latter quotes exemplify the participants who expressed having their three BPNs supported during the treatment and the journey they had from being rather extrinsically motivated to being more autonomously motivated for CBT and future dental attendance. The internalization process they went through made them more able to integrate and display a more high-quality autonomous motivation and behavior. Increased autonomy and self-regulation resulted in less DA, greater clarity of thought, a more positive rather than negative train of thought, finding their strengths, and better self-care. Collectively, these changes appeared to provide them with an improved quality of life.

## Discussion

This study aimed to explore patients’ experiences with CBT interventions delivered by TADA teams. Another objective was to apply SDT’s BPN framework [37–39] to analyze how participants experienced support for autonomy, competence, and relatedness during treatment, while also identifying which aspects of CBT they found beneficial. The participants reported better treatment effects and hopes of a future less characterized by DA and dental avoidance, along with an improved quality of life when they felt that their BPNs were supported. They linked their beneficial treatment effects to their positive relationships with the psychologist and dental personnel. They also reported having successfully managed their elevated levels of anxiety during CBT by using autonomous self-regulation and their inherent capacities. In turn, those newly obtained capabilities nurtured their plans for future dental visits and expectations of greater well-being.

Five themes emerged regarding patients’ perceptions of the CBT provided by the TADA teams.

### Theme 1: Establishing positive relationships with the psychologist and dental personnel

Our findings align with previous research [19, 25, 60, 61], which also emphasizes the importance of supportive and trusting clinical relationships within an unrushed clinic environment, particularly in trauma-informed settings like the TADA service, as essential for fostering relatedness and promoting effective treatment. The tendency for some patients in the TADA service to dissociate when CBT triggers trauma-related memories is in line with prior studies, which show that individuals with PTSD can re-experience trauma in therapeutic settings [19, 61]. Thus, taking a holistic interest in patients attending the TADA service and fostering their BPN satisfaction appears essential to helping them remain consciously engaged in treatment. This is also supported by Bryne et al. [62], who found that vulnerable patients in the TADA service valued an approach where they were viewed and treated as whole individuals. The importance of nurturing a trusting relationship between dentists and patients is supported by studies investigating the needs of individuals with DA and trauma histories [24, 63]. The participants’ emphasis on the value of an unrushed setting and active involvement in CBT aligns with previous SDT research, which highlights the importance of supporting patients’ BPNs in therapeutic contexts, blending insights from psychodynamic and humanistic traditions with evidence-based practices [64].

Participants described how the TADA team’s structured approach – clear goals, actionable guidance, and constructive feedback – supported their competence, mindfulness, reflections and engagement with treatment, aligning with earlier SDT research [65, 66]. This balance of structure and autonomy support seemed to promote effective anxiety management, increased energy, and more self-determined motivation. When participants experienced both autonomy support and structure, they often described developing better self-regulation, internalization of treatment goals, and stronger engagement, resonating with findings from SDT research [67, 68]. However, insufficient individualized, capacity-oriented interventions during CBT appeared to impede some participants’ fulfillment of their BPNs. These insights highlight the importance of integrating autonomy support with a suitable, individualized structure to promote lasting well-being, consistent with findings from previous research [66, 67].

### Theme 2: Experiencing enhanced competence in managing one’s anxiety

From the participants’ narratives, it was evident that learning to regulate their emotions and physiological reactions during CBT played an important role in becoming more mindful, authentic, and perceptive, especially when their anxiety levels became intolerable. This development seemed feasible because when patients signaled their concerns, the therapist demonstrated attentive listening. This approach nurtured their needs for competence, relatedness, and autonomy, leading to more authentic behavior, consistent with prior research [38, 69]. An authentic attitude aligns with autonomy support, and these patients often find it more appealing to stay in treatment due to its integration with their personal values and goals [38]. Conversely, individuals with a less authentic attitude may find high autonomy support challenging, as it can feel unfamiliar, potentially eliciting heightened DA [69]. This sense of discomfort may lead to feelings of abandonment or internal conflict. Providing choices in such cases can provoke anxiety, as these individuals may lack the integrated values and goals needed to make decisions. Research confirms that autonomy-supportive environments foster authenticity in patients [38, 70].

Our findings are consistent with previous studies underscoring that when individuals regulate emotions and behaviors in an autonomous and authentic manner, they are more likely to develop a meaningful sense of competence [39, 71]. Such high-quality motivation has been shown to enhance individuals’ ability to achieve desired health outcomes [72]. The insights shared by participants align with prior research grounded in SDT, which highlights that engaging with meaningful treatment rationales and confronting challenges in a present and reflective manner can support the development of self-regulation by satisfying the BPNs [55, 65, 73]. Meeting the BPNs has been shown to enhance treatment motivation and promote sustained behavioral change [38, 72, 74]. Participants who described feeling autonomously motivated also expressed that they were more able to persist through challenging tasks and integrate therapeutic recommendations. This contrasts with controlling treatment styles, which, as noted in SDT research, undermine autonomy, competence, and relatedness, often exacerbating DA [54, 75].

Some participants reported struggling to remain mindfully present during CBT, which they seemed to associate with limited development of emotional skills or anxiety-related competence. They tended to comply automatically with the therapist’s suggestions, without reflecting on their own needs or interests. This resulted in controlled motivation, with behavior regulated externally by the therapist. Consequently, these participants seemed to experience limited behavioral change compared to those with more autonomously regulated motivation. This aligns with previous research showing that patients with a history of continuous abuse may instinctively respond to perceived threats or controlling therapists with external, mechanical compliance or submission [76]. Dentists might misinterpret this compliance as agreement, highlighting the need for a trauma-sensitive approach tailored to support BPNs. Mindfulness training, which emphasizes autonomous self-regulation, authenticity, and competence building, can be particularly challenging for trauma patients prone to dissociation. Dental personnel would do well to be especially attentive to these challenges. In SDT-based psychotherapy, fostering mindful awareness has been shown to enhance integration, self-regulation, and personal satisfaction by addressing the BPNs [34, 64]. Moreover, fulfilling the three needs may reduce patients’ vulnerability and enhance their confidence in making effective decisions to navigate the environment [65].

Despite feeling more autonomously motivated in managing their DA during CBT, some participants struggled to take ownership of their treatment by expressing their preferences and acting in a self-directed and self-regulated manner. This may stem from insufficient trust and relatedness building, competence development, and unfulfilled autonomy needs. When fulfilled, these needs appeared to have enhanced autonomous motivation, helping most participants manage their anxiety effectively and with authentic personal investment. This was reported to contribute to improved well-being, consistent with previous research [34].

### Theme 3: Discovering the opportunity to use one’s own capacities

Participants noted that autonomy-supportive dental personnel appeared to unlock their inner capacities and self-knowledge, aligning with SDT research emphasizing the role of self-acceptance in reducing shame [38, 77, 78]. Reduced shame seemed to enable participants to embrace their emotions without self-judgment, fostering openness to growth, self-regulation, and active engagement in CBT. This appeared to support better anxiety management and confidence in treatment. These findings correspond with previous SDT research showing that when autonomy, competence, and relatedness are supported, particularly through warmth, empathy, and non-controlling communication, individuals are more likely to develop autonomous motivation and access their inner resources [38, 77]. By considering patients’ perspectives and acknowledging their past challenges, the dental personnel appeared to foster an autonomy-supportive environment. This approach likely contributed to participants remaining engaged in treatment, applying new skills, and internalizing effective behaviors, consistent with prior research [73]. These findings also align with those of Halvari et al. [79], who reported that autonomy support and need satisfaction in dental treatment were associated with lower DA and greater perceived competence and motivation, ultimately promoting better oral health behaviors.

During the interviews, some participants described feeling more autonomously motivated at the outset of CBT and gaining increased confidence in managing future DA in treatment settings. These participants also reported having clearer plans for maintaining regular dental visits. Their accounts suggested that their BPNs were supported, which may have contributed to their ability to recognize the value of CBT and engage more willingly in taking personal responsibility for regulating their behavior in a more autonomous manner. These findings are supported by previous research, which suggests that individuals who perceive CBT as personally meaningful are more likely to internalize and integrate more autonomous motivation and develop more effective strategies for regulating anxiety [34]. This is further supported by Pelletier et al. [80], who found that the more autonomously and volitionally patients engaged in therapy, the more attentive and satisfied they became, leading to a stronger intention to continue treatment. Similarly, research suggests that oral health professionals may find it easier to interact with patients who exhibit a high degree of autonomy orientation compared to those with lower autonomy orientation [55].

### Theme 4: Identifying factors nurturing plans for future dental visits

Creating a coping plan seemed to enhance participants’ confidence in future dental visits by fostering predictability, autonomy, and safety in the dentist-patient relationship. The significance of predictability is reinforced by recent findings among TADA patients, who reported reduced DA when practitioners provided gentle, patient-centered care along with clear, consistent communication in treatment [62]. When psychological needs were met during CBT, participants appeared more capable of managing increased DA, highlighting the value of a trusting, long-term relationship with a dentist for sustained self-regulation. Similar findings were reported by Kranstad et al. [24], who emphasized the importance of safe, enduring dentist-patient relationships for individuals with histories of sexual trauma. Although our findings revealed that the participants were satisfied with CBT, several expressed having external or introjected regulations (i.e. not autonomously self-regulated) and exhibited insecurity concerning their treatment results and how they would cope with future dental treatment settings. Many patients attending the TADA service may experience behavioral and mental health conditions marked by disruptions in autonomy [45]. Their behaviors, thoughts, and emotions may feel pressured, compelled, or controlled, or even uncontrollable, which corresponds with external or introjected regulations [45, 73, 81]. For instance, conditions such as anxiety disorders and depression can result in a lack of autonomous motivation, leaving patients either unable or amotivated to pursue personal goals. This aligns with evidence suggesting both external and internal pressures to behave in certain ways (i.e. introjected regulation) can deplete vitality and energy, whereas more autonomous forms of self-regulation tend to have the opposite effect [82]. In certain conditions, particularly those involving complex trauma (e.g. dissociative disorders), behaviors may occur without intentional self-regulation (i.e. external or introjected regulation) [45]. This lack of intentional behavior may hinder vulnerable TADA patients from engaging in effective self-regulation or developing and adhering to treatment plans, an important consideration when supporting this population.

### Theme 5: Experiencing additional effects on quality of life

Participants reported a range of quality-of-life improvements when their BPNs were nurtured during treatment. This aligns with findings from research, which emphasizes that autonomy-supportive healthcare environments promote psychological need satisfaction, contributing to internalization, positive behavioral change, and enhanced well-being [47]. Many participants described feeling more vital and mentally resilient, with a reduced tendency to be triggered by past traumas. This shift appeared to support their sense of competence, enabling them to express their authentic selves more clearly and envision a more positive future. Since vitality reflects the energy available to the self rather than mere activation, it is expected that fulfilling the BPNs will sustain or enhance vitality and support the self-regulatory capacities of the participants, consistent with prior research [82]. In line with SDT principles, most participants experienced reduced DA as their motivation shifted from externally controlled forms toward more autonomously regulated ones. These motivational shifts reflect increased autonomous self-regulation and engagement, which tend to occur when individuals feel that their psychological needs are supported. Such experiences underscore the potential benefits of integrating SDT-informed approaches into therapeutic settings, not only to improve specific treatment outcomes such as reduced DA, but also to promote broader personal growth, enhanced well-being, and improved quality of life. This broader impact is further supported by a recent CBT study in the field of oral health [83], which found that, in addition to reduced DA, participants also reported reductions in comorbid psychological symptoms, including anxiety, depression, and PTSD.

While many participants initially reported motivation to begin CBT, a factor consistently associated with positive psychotherapy outcomes [34], our findings revealed that autonomous motivation fluctuated throughout the course of treatment. This is consistent with previous research [34, 37], which highlights that patients often show resistance to change rather than entering therapy with fully autonomous self-regulated motivation. These findings underscore the importance of the therapist’s role in fostering autonomy support and creating a therapeutic environment that supports patients’ BPNs. Further understanding how CBT nurtures patients’ self-regulated and self-endorsed autonomous motivation, along with autonomy related to simultaneous competence development, the internalization process, and BPNs, seems important [34, 65]. Although CBT emphasizes the importance of rapport and volitional participation, the methods for achieving this are often unspecified [64]. In contrast, SDT emphasizes autonomy support through the development of appropriate autonomy, competence, and trusting relationships. It provides therapists with a comprehensive psychological framework grounded in evidence-based research, offering clear guidelines on fostering healthy, autonomous, and self-regulated motivation. Our findings show that participants felt more competent and capable of managing their DA when therapists acknowledged their emotions, provided meaningful choices, and offered constructive, non-judgmental feedback. These therapist behaviors were experienced as autonomy-supportive and appeared to foster more integrated autonomous motivation and self-regulation. This aligns with the findings of Williams et al. [72], who demonstrated that autonomy support facilitated the internalization of autonomous and competence-related motivation in diabetes self-management, leading to sustained behavioral change.

The findings of this study highlight key successes, some challenges, and considerations in applying CBT as a treatment method within dental treatment settings for trauma patients and those with severe DA. In outcome-oriented approaches like CBT, motivation is often considered a prerequisite for successful treatment [34]. In contrast, process-oriented approaches such as SDT view motivation as something to be nurtured by fostering self-determination and the development of more autonomous, self-regulated behavior during the therapeutic process [34]. Therefore, emphasizing patients’ autonomous and self-determined motivation by adopting autonomy-supportive approaches to address their BPNs during CBT can help patients achieve more meaningful and personally relevant treatment outcomes aligned with their integrated values. This, in turn, can enhance the effectiveness of the intervention. In this regard, SDT serves as a valuable theoretical framework for understanding more of the mechanisms at play and identifying the factors that may improve the efficacy of CBT. Our findings suggest that while most participants described supportive relationships with dental personnel, indicating a consistent fulfillment of the need for relatedness, there was greater variability in how autonomy and competence were experienced. Some participants reported difficulties in asserting their preferences (i.e. needs) or in fully understanding and managing their anxiety, which may indicate insufficient support for the needs of autonomy and competence. SDT posits [38], and empirical research confirms [55, 64, 84], that optimal outcomes more likely arise when therapeutic environments are autonomy-supportive. Therefore, it may be beneficial for dental practitioners, particularly those integrating CBT into care and working with patients with severe DA or trauma histories, to receive additional training in SDT-informed approaches. Such training could help clinicians more systematically support psychological needs.

These insights highlight the potential value of optimizing both the content and delivery of sessions, with SDT as a guiding framework. Facilitating patients’ autonomy, beyond simply respecting it, is both therapeutically beneficial and an ethical responsibility for therapists. Autonomy is essential to human agency and well-being, as it enables patients to make more self-informed decisions about whether to pursue change or postpone their efforts, thereby empowering them to take ownership of their treatment process [34]. As emphasized in previous research, providing such support helps patients develop and exercise context-appropriate autonomy, particularly in challenging health care settings [34]. Furthermore, competence is strengthened when autonomy is supported. When patients make choices for themselves in an autonomous way, they are more likely to align these choices with their perceived competence and personal values. As a result, their sense of competence becomes more relevant and meaningful. Such facilitation may increase the likelihood of more patients experiencing self-determined, autonomous motivation (i.e. identified or integrated regulation) during CBT, potentially fostering stronger competence and sustained behavioral change in regulating DA. Additionally, this approach may contribute to an improved quality of life.

This study underscores the importance of patients’ perspectives on the benefits of working with autonomy-supportive therapists who integrate mindfulness and provide structured treatment with optimal challenges.

### Strengths and limitations

The study did not include the experiences of torture victims. However, the sample was diverse in terms of age, gender, cultural background, education, and experiences with trauma and dental treatment. Participants’ gratitude for the TADA service may have introduced positive bias, and recall bias could have influenced their accounts as experiences were shared retrospectively. To minimize this, the interviews were conducted shortly after participants completed the CBT phase of the TADA service. Consistency in elicitation and interpretation techniques across interviews was ensured by using the same interviewer [85]. The interviewer had relevant experience from working as part of a TADA team at another clinic, which provided familiarity with the subject but also carried potential risks of subjective bias. To address this, data analysis involved multiple authors to balance perspectives. Further research is needed to explore how these findings apply to a broader population of patients attending the TADA service, as the results of this qualitative research are not generalizable.

## Conclusion

Integrating evidence-based insights from SDT into CBT can help therapists systematically foster autonomous, self-regulated motivation through the internalization process, while clarifying the elements that drive behavioral change and support patients in regulating anxiety. SDT and CBT may complement each other in enhancing treatment effectiveness, personal development, mental health, and quality of life in this vulnerable patient group. Based on our findings, we recommend that the TADA service more explicitly adopt SDT as a guiding framework by building on its autonomy-supportive practices to better support patients’ BPNs.
